# Multipath Effects in Millimetre-Wave Wireless Communication using Orbital Angular Momentum Multiplexing

**DOI:** 10.1038/srep33482

**Published:** 2016-09-23

**Authors:** Yan Yan, Long Li, Guodong Xie, Changjing Bao, Peicheng Liao, Hao Huang, Yongxiong Ren, Nisar Ahmed, Zhe Zhao, Zhe Wang, Nima Ashrafi, Solyman Ashrafi, Shilpa Talwar, Soji Sajuyigbe, Moshe Tur, Andreas F. Molisch, Alan E. Willner

**Affiliations:** 1 Department of Electrical Engineering, University of Southern California, Los Angeles, CA 90089, USA; 2 NxGen Partners, Dallas, TX 75219, USA; 3 University of Texas at Dallas, Richardson, TX, 75080, USA; 4 Intel Labs, Intel Corporation, Santa Clara, 95054, USA; 5 School of Electrical Engineering, Tel Aviv University, Ramat Aviv, 69978, Israel

## Abstract

Electromagnetic waves carrying orbital angular momentum (OAM) have been used for mode division multiplexing in free-space communication systems to increase both the capacity and the spectral efficiency. In the case of conventional wireless communication links using non-OAM beams, multipath effects caused by beam spreading and reflection from the surrounding objects affect the system performance. This paper presents the results of analysis, simulations, and measurements of multipath effects in a millimetre-wave communication link using OAM multiplexing at 28 GHz. Multipath-induced intra- and inter-channel crosstalk, which are caused by specular reflection from a plane parallel to the propagation path, are analysed and measured. Both the simulation and the experimental results show that an OAM channel with a high OAM number *ℓ* tends to suffer from both strong intra-channel crosstalk and strong inter-channel crosstalk with other OAM channels. Results of the analysis show that this observation can be explained on the basis of both the properties of OAM beam divergence and the filtering effect at the receiver, which is associated with the spiral wavefront of OAM beams.

There are many compelling applications for high-spectral-efficiency and high-transmission-capacity point-to-point free-space line-of-sight (LoS) communication links, including indoor, data centre, front-haul, and back-haul systems^1^,^2^. Conventional spatial multiplexing requires multiple spatially separated aperture pairs and crosstalk mitigation through digital signal processing (DSP)^3^. However, another approach to increase capacity and spectral efficiency involves the use of a set of spatially orthogonal modes propagating through a single aperture pair. These spatially orthogonal beams can be efficiently multiplexed at the transmitter end, spatially overlapped during propagation, and efficiently demultiplexed at the receiver end, with low inherent crosstalk. The orthogonality of the modes can greatly reduce the complexity of signal post processing. One method to implement such multiplexing is orbital angular momentum (OAM) multiplexing^4^,^5^,^6^,^7^,^8^. An OAM beam exhibits phase-front ‘twisting’ in a helical manner as it propagates^9^. The OAM value *ℓ* is defined by the continuous phase change from 0 to 2π*ℓ* of the wavefront in the angular direction, and OAM beams with distinct OAM orders are spatially orthogonal to one another. Beams with different values of *ℓ* can form a set of orthogonal beams, where each vortex beam exhibits a ring-like intensity structure. Therefore, OAM enables efficient multiplexing and demultiplexing and facilitates the simultaneous propagation of multiple beams carrying independent data streams through a single pair of aperture (one transmitter aperture emitting multiple OAM modes and one receiver aperture receiving multiple OAM modes), with low inherent crosstalk. The total data rate and spectral efficiency can be increased on the basis of the number of multiplexed OAM channels, and the complexity of signal post processing could be reduced due to the low inherent crosstalk.

As in the case of all wireless communication systems, multipath effects^3^,^10^,^11^,^12^,^13^,^14^ are likely to have significant effects on OAM multiplexing systems, particularly since the divergence of OAM beams with *ℓ ≠* 0 is greater than that of conventional beams with *ℓ = *0 (e.g. Gaussian beams)^15^. Several unique factors associated with an OAM-multiplexed link present the following interesting technical challenges: (1) ***Intra- and inter-channel crosstalk***: The reflected energy can be coupled not only into the same data channel of the same OAM value (i.e. as in the case of a conventional single beam link) but also into another data channel with a different OAM value. Therefore, both intra- and inter-channel crosstalk can occur. (2) ***Beam intensity and phase***: An OAM beam has a doughnut-shaped intensity profile showing low power in the centre and a peak showing maximum power around a ring; further, the beam exhibits an azimuthal phase change with a value of 2π*ℓ*. In addition, the detection of a specific OAM beam requires a spatial filter to filter out energy on the other beams, which may reduce the received power from the reflected beam.

In this study, we analyse the multipath effects of OAM channels caused by specular reflection from a surface parallel to the communication link. We show that such reflection causes the distortion of the helical wavefront of the received OAM beams, leading to multipath-induced intra-channel and inter-channel crosstalk. The OAM beam divergence and the spatial filtering effect of the OAM receiver are found to determine the crosstalk. Results of the analysis and measurement show that OAM channels with high values of *ℓ* tend to suffer from both strong intra-channel crosstalk and strong inter-channel crosstalk with other channels.

## Concept of Multipath-Effect-Induced Intra- and Inter-Channel Crosstalk of OAM Channels

**Figure 1:**
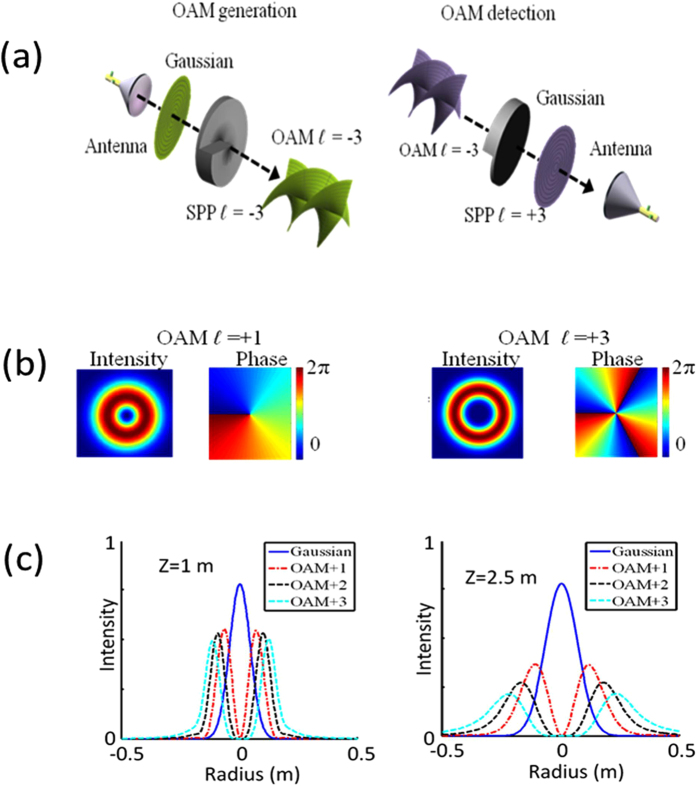
(**a**) Intensity and wavefront phase of OAM beams with *ℓ* = +1 and *ℓ* = +3. (**b**) Generation and detection of an OAM beam. (**c**) 1-D beam intensity of OAM beams at distances of 1 m and 2.5 m.

One approach to generate and detect OAM millimetre-wave beams involves the use of spiral surface plates (SPPs), as shown in Fig. 1(a). At the transmitter end, a millimetre-wave OAM beam can be generated by propagating a beam with *ℓ* = 0 through an SPP with an OAM number *ℓ*^7^. At the receiver end, another SPP with *−ℓ* can be used to convert the OAM beam back to be a beam with *ℓ* = 0 for detection. Figure 1(b) shows the ring-shaped intensity and spiral phase of the OAM beam wavefront with *ℓ* = +1 and *ℓ* = +3. It should be noted that given the same size of the initial beam of *ℓ* = 0, OAM beams passing thought the SPPs with a higher value of *ℓ* will diverge faster. Figure 1(c) shows the simulation result of the one-dimensional (1-D) intensity profile of OAM beams with different values of *ℓ* at propagation distances of 1 m and 2.5 m. The frequency of the millimetre wave is 28 GHz, and Gaussian beams (*ℓ = *0) are emitted from the lensed horn antenna with a diameter of 15 cm. In the simulation we assume the Gaussian beam has a beam width of 7.5 cm. The diameter of the SPP is 30 cm. It is noted that mm-wave frequency range is usually defined from 30 GHz–300 GHz. However, in wireless communications, a 28 GHz carrier frequency is also considered as mm-wave communications^16^. From this figure, it is observed that OAM beams with a higher value of *ℓ* diverge more as they propagate and their intensity profile shows low power in the centre.

**Figure 2:**
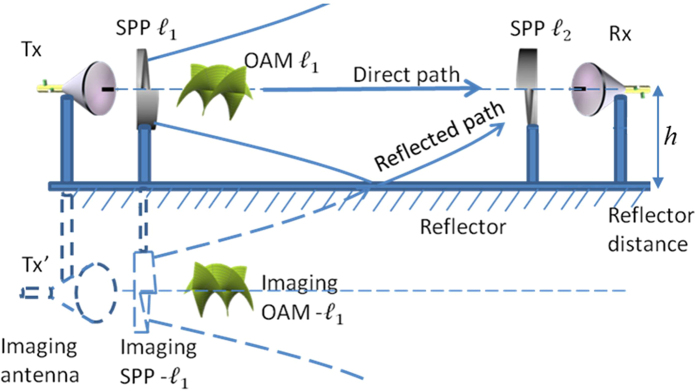
Multipath effects of an OAM channel caused by specular reflection from a parallel ideal reflector. Owing to beam divergence, a part of the OAM beam will be reflected. The reflected OAM beam can be observed as an off-axis OAM beam from the imaging antenna Tx′ and the imaging SPP′ with an opposite OAM number.

Figure 2 shows the concept of the multipath effects of an OAM beam caused by the specular reflection from a reflector parallel to the link. An OAM channel with an OAM number of *ℓ*_*1*_ is transmitted along the link. At the receiver end, an SPP with an OAM number of *ℓ*_*2*_ and an antenna is used to receive the OAM channel with an OAM number of *ℓ*_*2*_. Ideally, power can be recovered only when *ℓ*_*1*_ = *ℓ*_*2*_ owing to the orthogonality of OAM beams in a line-of-sight link. However, the orthogonality no longer holds when the receiver receives the reflected beam. As shown in Fig. 2, a reflector is placed at a distance of *h* away from the beam centre. Assuming that the reflector has a reflection coefficient of 100%, the reflected beam can be observed as an OAM beam from an imaging antenna Tx′ and an imaging SPP with an OAM number of *−ℓ*_*1*_ (reflection changes the sign of the OAM value). Therefore, the receiver will receive an OAM beam with an OAM number of *ℓ*_*1*_ from the original link as well as a reflected beam with an OAM number of *−ℓ*_*1*_ from an offset link, which is placed at a distance of 2*h*.

**Figure 3:**
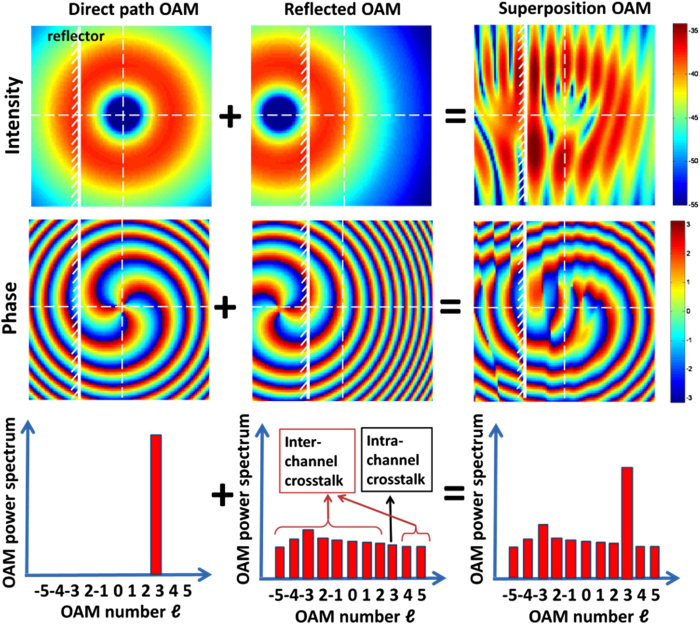
Simulation results showing the intensity, phase, and OAM spectrum of the direct path OAM beam, reflected path OAM beam, and the actual beam at the receiver. The actual beam is the superposition of the direct OAM and the reflected OAM beams. Because the reflected OAM beam is along the off-axis, it is no longer orthogonal to the OAM beams in the direct path and is likely to cause both inter-channel and intra-channel crosstalk at the receiver. The white solid line represents the reflector.

Orthogonality of the OAM beams depends on the spiral wavefront. Reflection is likely to distort the wavefront phase and induce both intra-channel and inter-channel crosstalk. Further, reflection causes the distortion of the OAM beam intensity, giving rise to both intra-channel and inter-channel crosstalk. To illustrate this phenomenon, we use an OAM beam with *ℓ*_1_ = +3 as an example. In Fig. 3, the left-hand-side column shows the intensity, phase, and OAM spectrum of the OAM beam in the direct path. The entire power is in the OAM state of *ℓ*_1_ = +3. The middle column shows the reflected OAM beam. The reflected OAM beam exhibits an OAM number of *ℓ*_1_′ = −3, and it is offset to the direct link. As a result, when the reflected OAM beam is decomposed with respect to the OAM basis along the direct path axis, power diverges onto a wide range of OAM states, leading to intra-channel crosstalk with an OAM channel with *ℓ*_1_ = +3 and inter-channel crosstalk with the other OAM channels with *ℓ*_1 _≠ +3^17^. The right-hand-side column in Fig. 3 shows the actual beam at the receiver, which is the superposition of the direct and the reflected beams. The intensity exhibits a fringing pattern owing to the interference between the direct and the reflected beams. The wavefront phase is also distorted owing to the multipath effect. The power of the actual received OAM with *ℓ*_1_ = +3 differs from the one of the directed path because of the intra-channel crosstalk from the reflected beam, and the received power of the other OAM beams with values of *ℓ*_1_ ≠ +3 is nothing but the inter-channel crosstalk from the reflected beam.

**Figure 4:**
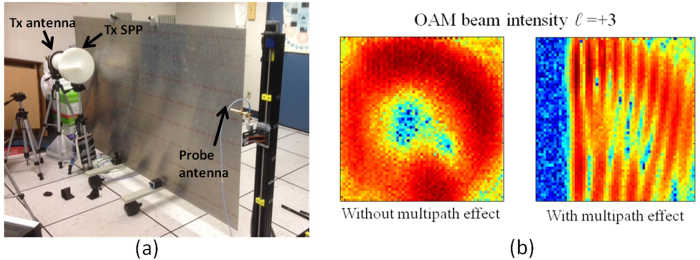
Experimental setup for investigating the multipath effects of OAM channels. A movable aluminium sheet is used as an ideal reflector, which is placed parallel to the propagation path. (**b**) Intensity of the OAM beam with *ℓ* = +3 without and with the multipath effects.

The experimental setup for investigating the multipath effects of OAM channels is shown in Fig. 4(a). An aluminium sheet with an area of 2.5 m × 1.5 m is mounted on a cart and placed parallel to the link. The distance between the path and the reflector can be varied by moving the cart. It is observed that the multipath effect becomes stronger with a decrease in the distance between the path and the reflector. As an example, Fig. 4(b) shows the intensity of an OAM beam with *ℓ* = +3 in the absence of a reflector, as well as the intensity of the OAM beam when the reflector is placed close to the link. From this figure, it is observed that the measured fringing intensity pattern caused by the interference of the direct and the reflected beams resembles the simulation result shown in Fig. 3.

In the experiment, the lensed horn antennae have a diameter of 15 cm and gain of ~30 dBi. The 2.5 m link loss for *ℓ* = 0, +1 and +3 is measured to be around 20 dB, 29 dB and 46 dB. When two channels *ℓ* = +1 and *ℓ* = +3 are multiplexed, the received power for both channels is around −30 dBm and they have the same level of SNR. Therefore the transmitter power is −1 dBm for *ℓ* = +1 channel and 16 dBm for *ℓ* = +3 channel.

### Multipath Effect on a Single OAM Channel and Intra-Channel Crosstalk

**Figure 5:**
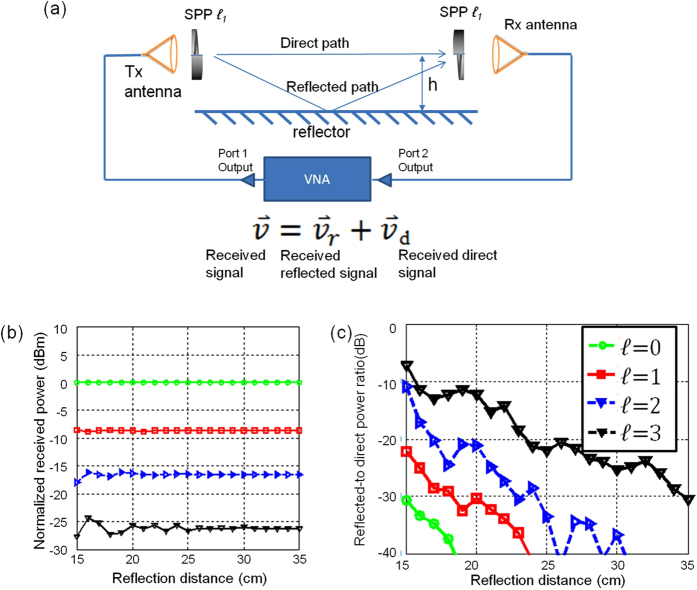
Setup to study intra-channel crosstalk of a single OAM channel. (**b**) Experimental results of the received power OAM beams with different values of *ℓ* as functions of the reflector distance h. (**c**) Experimental results of the reflected-to-direct power ratio for OAM beams with different values of *ℓ*.

We first studied the intra-channel crosstalk induced by the multipath effect. This intra-channel crosstalk is shown in Fig. 5(a), where only an OAM channel with an OAM number of *ℓ*_1_ is transmitted and received. A vector network analyser (VNA) is used to generate and detect a 28-GHz millimetre-wave signal. Two sets of lens horn antennae and SPPs with an OAM number of *ℓ*_1_ are used on both sides to generate and detect OAM beams. According to the abovementioned multipath model, the received signal 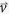 can be analysed in terms of the addition of signal 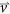
_d_ from the direct path and 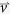
_r_ from the reflected path; the relationship among these signals is expressed as 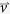
=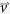
_r_ + 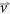
_d_. Figure 5(b) shows the experimental results of the normalised received power as a function of the reflector distance *h* for OAM beams with different values of *ℓ*. As shown in this figure, the following two effects are observed. First, for a high value of *ℓ*, the received signal power |
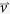
|^2^ is low, which can be explained on the basis of the divergence property of OAM beams^18^. Second, when the reflector distance *h* is small, the received power |
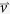
|^2^ fluctuates with a change in the reflector distance, which is explained in terms of the constructive or destructive interference between the signals from the direct and reflected paths^3^. Moreover, the higher the value of *ℓ*, the larger is the fluctuation in power, indicating stronger multipath effects for OAM channels with higher values of *ℓ*.

To verify the abovementioned conjecture, we define |
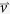
_r_|^2^/|
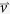
_d_|^2^ as the reflected-to-direct power ratio of the OAM channels, and we calculate this value by measuring 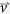 and 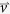
_d_ with the VNA. 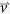
_d_ is measured when the reflector is removed from the link. Further, 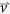 is measured when the reflector is included in the setup. Then, 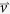
_r_ can be obtained from 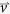
_r_ = 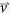 − 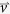
_d_. Figure 5(c) shows the measured reflected-to-direct power ratios as functions of the reflector distance for OAM beams with different values of *ℓ* ranging from 0 to 3. The result shows that an OAM channel with a higher value of *ℓ* exhibits a higher reflected-to-direct power ratio.

A theoretical expression of the displaced LG beam can be found in reference^19^, which may lead to some theoretical expression of intra- and inter-crosstalk under certain ideal assumptions. However, it is hard to directly apply the mathematical equations in the analysis of a more practical mm-wave OAM communication link. The reason is that OAM beams are not always LG beams, which is the case in our experiment, as well as the diffraction effects caused by the finite aperture at both transmitter and receiver sides. Therefore, we use a numerical simulation tool called Fresnel diffraction^20^ to simulate the propagation and reflection of OAM beams, and use the theory of OAM decomposition^21^ to characterize the inter- and intra-crosstalk in the simulation. In the experiment we take measurements in a specific setup with fixed propagation distance, aperture size, two OAM values *ℓ* = +1 and *ℓ* = +3 and a moveable reflector which is parallel to the link to verify the simulation results.

**Figure 6:**
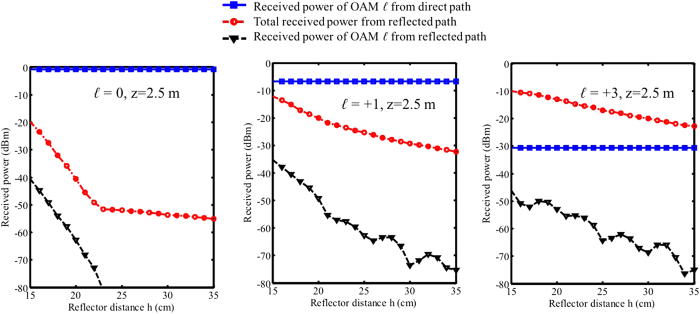
Simulation results of intra-channel crosstalk for OAM beams with *ℓ* = 0, *ℓ* = +1, and *ℓ* = +3 as functions of reflector distance h when propagation distance z = 2.5 m. Blue-squared curves: received power of OAM channel *ℓ* from the direct path in the receiver aperture. Red-circled line: total power collected by the receiver aperture from the reflected path. Black triangle line: received power of OAM channel *ℓ* from the reflected path. The difference between the black triangle curve and the blue-squared curve denotes the intra-crosstalk level.

The simulation results shown in Fig. 6(a,b) provide a more detailed analysis for OAM intra-channel crosstalk. In Fig. 6(a) we show the received power of directed path and reflected path as functions of the reflector distance h. The blue lines show the received power of an OAM channel where *ℓ* = *ℓ*_1_ from the direct path, which is collected by the receiver aperture. The power from the direct path is found to decrease with an increase in the OAM number *ℓ*_1_; this change is explained on the basis of the divergence characteristics of OAM beams. The red lines show the total power collected by the receiver aperture from the reflection path. The total power from the reflected path increases with the OAM number, because for a high value of *ℓ*, the OAM beam spreads faster so that more energy gets reflected into the receiving aperture. The black line shows the received power from the reflection path that only belongs to the OAM channel with *ℓ* = *ℓ*_1_, which indicates the absolute intra-channel crosstalk from the reflected path. It should be noted that although the total collected power (red curves) from the reflected path increases significantly with the OAM number *ℓ*, the received power of the specific OAM channel (black curves) for different OAM numbers is not that much different. This effect can be qualitatively explained as follows.

Only a part of the reflected power belongs to the OAM channel with an OAM number of *ℓ*_1_. To determine this amount of energy, we calculate the overlap integral between the fields of the received reflected beam and the OAM beam of *ℓ*_1_. This calculation method is known as OAM decomposition^22^.


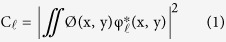


This formula is valid under the paraxial approximation. We use a scalar model here since the polarization of the reflected beam is as same as the direct beam’s polarization in this experiment. In a more general case the vectorial model needs to be considered if the reflection changes the polarization state. In the above equation, Ø(x, y) is the complex amplitude of the reflected beam in the receiver aperture, and φ_*ℓ*_(x, y) denotes the complex amplitude of the OAM beam in the receiver aperture. Given that a higher value of *ℓ* results in a more rapid change in the azimuthal phase of φ_*ℓ*_(x, y) and that the phase of Ø(x, y) is relatively uniform (because the reflected beam is off-centred, see Fig. 3), the value of the overlap integral C_*ℓ*_ is expected to be low owing to the averaging effect in the overlapped integral. This observation explains the reason for an increase in the distance between the red curves (total received power within receiving aperture) and the black curves (received OAM power C_*ℓ*_) with an increase in the OAM value.

On the other hand, it is observed that owing to beam divergence, power from the direct path decreases and the total collected reflected power increases with *ℓ*. Moreover, the phase structure of the receiver SPP helps reduce the intra-channel crosstalk from the reflected channel. The overall intra-channel crosstalk is, in fact, the difference between the black and the blue curves, which decrease with the value of *ℓ*, indicating strong intra-channel crosstalk for OAM beams with higher values of *ℓ*.

**Figure 7:**
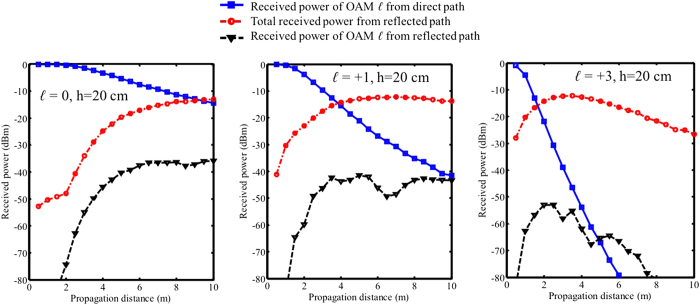
Simulation results of intra-channel crosstalk for OAM beams with *ℓ* = 0, *ℓ* = +1, and *ℓ* = +3 as functions of propagation distance z when reflector distance h = 25 cm. Blue-squared curves: received power of OAM channel *ℓ* from the direct path in the receiver aperture. Red-circled line: total power collected by the receiver aperture from the reflected path. Black triangle line: received power of OAM channel *ℓ* from the reflected path. The difference between the black triangle curve and the blue-squared curve denotes the intra-crosstalk level.

In Fig. 7, we show simulation results of the received power from direct and reflected paths as functions of propagation distance z when the reflector distance h = 25 cm. The blue curves show the received power from the direct path drops more rapidly for a higher *ℓ* value due to the divergence property that there is less power in the centre. Another effect is that for a higher *ℓ* value, the received reflected power is higher at a shorter distance. That can also be explained by the divergence property of OAM beams that the beam spread more rapidly and get reflected at a shorter distance. For *ℓ* = +1 and *ℓ* = +3, it is observed that the reflected power starts decreasing after certain distances. As illustrated in Figs 1 and 2, since the reflected OAM beam’s peak intensity changes at different distance, when the receiver is moved along the propagation distance it will experience the intensity change and see the highest power at certain distance where the intensity peak of the reflected beam locates. The overall effect, which is shown by the the difference between the black curve and blue curve, is that for a higher *ℓ* value, the same level of intra-channel crosstalk happens at a shorter distance. In Figs 6 and 7, at some points the curves show quick changes of the slope. We believe that this is due to the EM field truncation.

**Figure 8:**
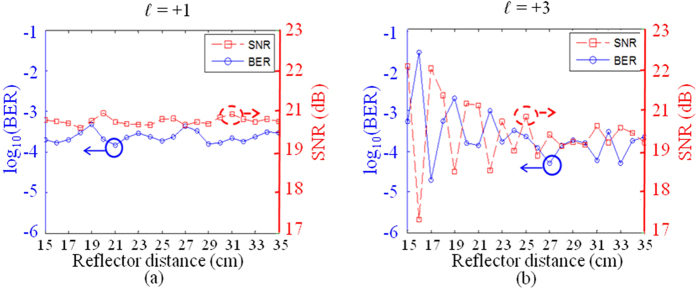
Measured BER and SNR as functions of the reflector distance *h* for (**a**) *ℓ* = +1 and (**b**) *ℓ* = +3. Stronger fluctuations of BER and SNR are observed for OAM with *ℓ* = +3 because of the stronger intra-channel crosstalk induced by multipath effects. The distance between Tx and Rx aperture is 2.5 m.

Next, we investigate the manner in which multipath-induced intra-channel crosstalk affects the performance of a single OAM channel. A 16-QAM signal is used with a data rate of 1 Gbaud. The reflection board distance is from 15 cm to 35 cm in the experiment (starts at 15 cm since this is the radius of Tx/Rx apertures). As shown in Figs 5 and 6, significant multipath effects are observed when the reflector distance is within 35 cm, corresponding to a distance difference of 1.7 cm~9.6 cm (a time delay of 55 ps~322 ps) between the direct and the reflected paths, which is estimated from the geometry shown in Fig. 2. Given that the wavelength is 1.07 cm for the 28-GHz carrier frequency, the signal-to-noise ratio (SNR) variation with the reflection distance *h* is expected owing to the constructive or destructive interference. The signal symbol is 30 cm in length (1 ns in time), which is much longer than the multipath difference; therefore, in this experiment, the inter-symbol interference effect is considered to be weak. Figure 8 shows the SNR and the bit error rate (BER) as functions of the reflector distance *h* for OAM channels with *ℓ* = +1 and *ℓ* = +3, respectively. When *ℓ* = +1, given that the intra-channel crosstalk is relatively low, moderate SNR and BER variations are observed. When *ℓ* = +3, significantly stronger fluctuations of the SNR and BER are observed owing to the stronger inter-channel crosstalk. The results show that the performance of an OAM channel with a higher value of *ℓ* is more affected by the intra-channel crosstalk.

### Multipath Effect of Multiplexed OAM Channels and Inter-Channel Crosstalk

**Figure 9:**
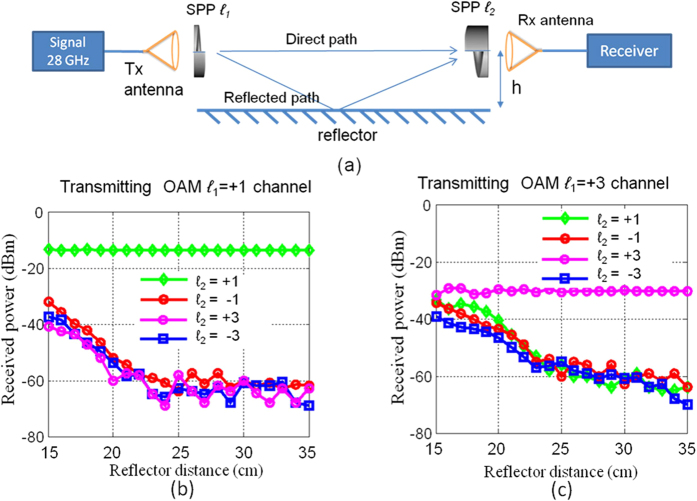
(**a**) Experimental setup to study the inter-channel crosstalk of OAM channels. (**b**) Power received from different OAM channels when the transmitted OAM number is *ℓ* = +1. (**c**) Power received from different OAM channels when the transmitted OAM number is *ℓ* = +3.

Inter-channel crosstalk is measured when the transmitter SPP OAM number *ℓ*_*1*_ is fixed and the receiver SPP OAM number *ℓ*_*2*_ takes different values. Figure 9(a) shows the experimental setup. A spectrum analyser is used to measure the amount of power that is received from the OAM channel *ℓ*_*1*_ when the SPP with OAM number *ℓ*_*2*_ is used at the receiver. Figure 9(b,c) show the results of the measurement of the received power of the OAM channels with OAM number *ℓ*_*2*_ as functions of the reflector distance *h* when *ℓ*_*1*_ = +1 and *ℓ*_1_ = +3, respectively. From these figures, it is observed that with an increase in *h*, the power received from the other OAM channels increases owing to the strong multipath effects. When *ℓ*_*1*_ = +1, the received power difference between *ℓ*_*2*_ = +1 and the other channels is >20 dB, indicating that the inter-channel crosstalk from the channel with *ℓ* = +1 to the other channels is less than 20 dB. In the case of the OAM channel with *ℓ*_*1*_ = +3, the inter-channel crosstalk from the channel with *ℓ* = +3 to the other channels is 2~9 dB.

**Figure 10:**
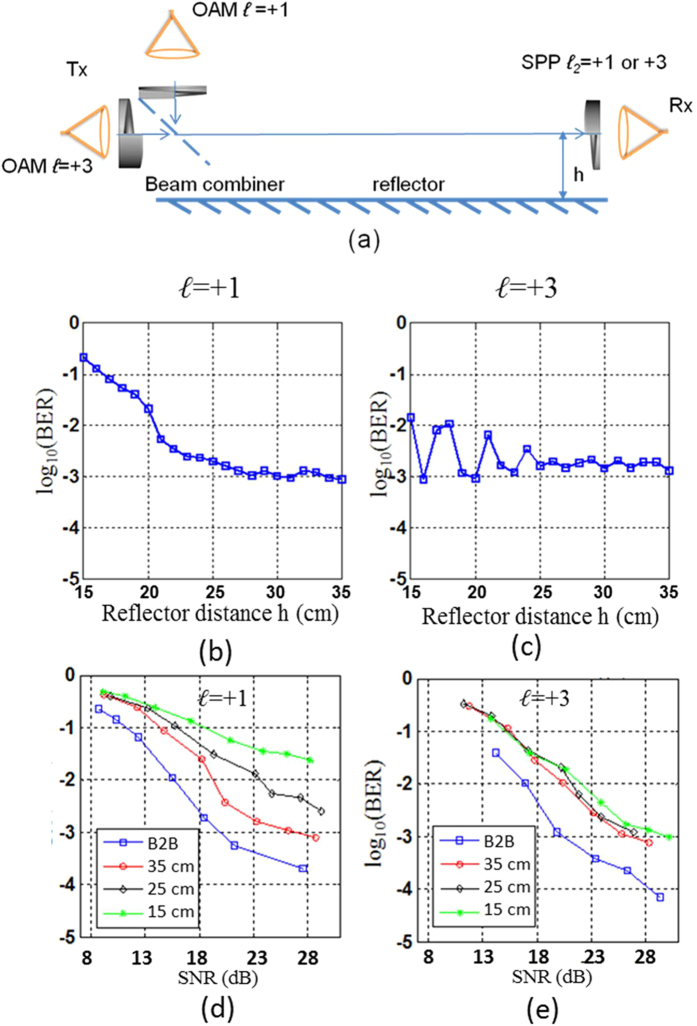
(**a**) Multipath effects in an OAM multiplexing communication link suffering from both the intra- and the inter-channel crosstalk induced by multipath effects. (**b**) Measured BER as a function of the reflector distance for the *ℓ* = +1 channel. (**c**) Measured BER as a function of the reflector distance for the *ℓ* = +3. (**d**) Measured BER as a function of the SNR for the *ℓ* = +1 channel. (**e**) Measured BER as a function of the SNR for the *ℓ* = +3.

Next we study the channel performance when two OAM channels with *ℓ* = +1 and *ℓ* = +3 are multiplexed as shown in Fig. 10(a). Two OAM channels are combined at the transmitter by using a beam combiner. At the receiver, one of the two OAM channels is detected by using the corresponding SPP. In this case, both the intra-channel and the inter-channel crosstalk exist because of the multipath effects. The signal on each OAM channel is a 1-Gbaud 16-QAM signal.

Figure 10(b,c) show the BERs as functions of the reflector distance *h* for *ℓ* = +1 and *ℓ* = +3, respectively (the SNR for both channels is ~22 dB in the absence of a reflector). The BER of an OAM channel with *ℓ* = +1 significantly increases when the reflector is close to the link. On the basis of the results of the previous intra- and inter-channel crosstalk measurement, we believe that a high BER is mostly caused by the inter-channel crosstalk from an OAM channel with *ℓ* = +3. Figure 8(c) shows a similar BER variation, as observed in Fig. 8(b), for the OAM channel with *ℓ* = +3. Given that the measured crosstalk from the OAM channel with *ℓ* = +1 to *ℓ* = +3 is low, we believe that the intra-channel crosstalk mainly contributes to the BER variation of the OAM channel with *ℓ* = +3. The results shown in Fig. 10(d,e) show the BER as functions of the SNR for channels with *ℓ* = +1 and *ℓ* = +3 at different reflector distances *h*. From the BER-SNR curves we can observe that in the case of the channel with *ℓ* = +1, the BER error floor increases from ~1 × 10^−3^ to ~1 × 10^−2^ and the power penalty compared to back-to-back (B2B) also increases when the reflector distance *h* decreases. This clearly indicates that the *ℓ* = +1 channel received more crosstalk from the *ℓ* = +3 channel when the multipath effects were stronger. In the case of the *ℓ* = +3 channel, however, we observe that the BER-SNR curves at different distances are quite similar (less than 2 dB power penalty between each other). This observation indicates that the inter-channel crosstalk from the *ℓ* = +1 channel is low and has limited influence on the performance of the *ℓ* = +3 channel.

## Discussion

In summary, we investigated the multipath-induced intra- and inter-channel crosstalk effects in a millimetre-wave communications link using OAM multiplexing. Due to the unique intensity and phase structure of OAM beams, the analysis is different from the traditional multipath effects. The multipath OAM channels with higher values tend to have stronger intra-channel crosstalk. In the case of OAM beams with higher *ℓ* values, a low amount of power will be received from the direct path. Although more power is collected by the receiver aperture, the receiver SPP serves as a filter and helps reduce the power from the reflected path. OAM channels with higher *ℓ* values also cause an increase in the inter-channel crosstalk with the other OAM channels in the presence of multipath effects. The results of our investigation primarily focus on the fundamental effect of multipath in an OAM multiplexing scenario and thus consider a single specular reflector. This scenario is not only practically relevant as being similar to a ground/wall reflection but also provides insights into the interaction between the direct and the reflected components. Further work may investigate the combined impact of multiple reflectors and diffused reflectors and diffused reflection, and explore using digital signal processing (such as using an equalizer) to mitigate multipath effects^23^.

## Additional Information

**How to cite this article**: Yan, Y. *et al*. Multipath Effects in Millimetre-Wave Wireless Communication using Orbital Angular Momentum Multiplexing. *Sci. Rep*. **6**, 33482; doi: 10.1038/srep33482 (2016).
